# A Community-Based Study to Estimate the Seroprevalence of Trichinellosis and Echinococcosis in the Roma and Non-Roma Population of Slovakia

**DOI:** 10.3390/ijerph15020251

**Published:** 2018-02-02

**Authors:** Daniela Antolová, Monika Halánová, Martin Janičko, Peter Jarčuška, Katarína Reiterová, Júlia Jarošová, Andrea Madarasová Gecková, Daniel Pella, Sylvia Dražilová, HepaMeta Team

**Affiliations:** 1 Department of Parasitic Diseases, Institute of Parasitology SAS, Hlinkova 3, 040 01 Košice, Slovakia; reiter@saske.sk (K.R.); jarosova@saske.sk (J.J.); 2 Faculty of Medicine, P. J. Šafárik University in Košice, 040 11 Košice, Slovakia; monika.halanova@upjs.sk (M.H.); martin.janicko@gmail.com (M.J.); peter.jarcuska@upjs.sk (P.J.); andrea.geckova@upjs.sk (A.M.G.); daniel.pella@upjs.sk (D.P.); 3 Department of Internal Medicine, Hospital Poprad, 058 01 Poprad, Slovakia; drazilova.s@nemocnicapp.sk

**Keywords:** *Trichinella* spp., *Echinococcus multilocularis*, *Echinococcus granulosus*, Roma population, Slovakia

## Abstract

Trichinellosis and cystic and alveolar echinococcosis are serious parasitic diseases transmissible between animals and humans. Moreover, alveolar echinococcosis is considered one of the most dangerous of human helminthoses. Roma communities are particularly numerous in Central and Eastern Europe. They are often concentrated in economically undeveloped regions and live in segregated localities with unsatisfactory housing and sanitary conditions. The study aimed to find out the seroprevalence of *Trichinella* and *Echinococcus* infections in the Roma population of segregated settlements and to compare it with the seropositivity of the non-Roma population of eastern Slovakia. Out of 823 samples, three sera showed seropositivity to *Trichinella* in the ELISA (Enzyme-linked immunosorbent assay) test. Subsequent Western blot reaction (WB) confirmed seropositivity in two Roma women. ELISA seropositivity to *E. multilocularis* was recorded in six persons (0.73%), and five (0.61%) respondents were seropositive to *E. granulosus*, but WB confirmed the presence of antibodies to *Echinococcus* spp. in one Roma participant. Positive persons suffered from unspecific clinical symptoms; *Trichinella*-positive persons reported headache, cough, fatigue, and muscle pain. The *Echinococcus*-positive participant suffered from headache and back pain. The study showed that the worse living conditions of the Roma community did not significantly influence the occurrence of *Trichinella* and *Echinococcus* infections in this minority.

## 1. Introduction

Trichinellosis, a parasitic disease with world-wide distribution is transmissible between animals and humans, in which it may cause serious health problems. Red foxes and wild boars are considered reservoirs of the infection in wildlife. Humans acquire the infection after the consumption of raw or undercooked meat of infected animals. Hunting activities connected with improper handling of meat and offal can increase the risk of the infection spreading into the domestic cycle and consequently, the risk of human infection [1]. In Slovakia, trichinellosis occurs primarily in wildlife, but human cases as well as sporadic outbreaks of the disease have also been recorded [2,3].

Alveolar and cystic echinococcosis, serious parasitic diseases caused by the larval stages of *Echinococcus multilocularis* and *E. granulosus* sensu lato (s. l), have been diagnosed in Slovakia for a long time. The infection arises after the accidental ingestion of infective eggs from the contaminated environment (soil, water, food) and causes serious health problems connected primarily with effects on the liver [4,5].

Roma are an ethnic minority living in many countries throughout the world, but they are particularly numerous in Central and Eastern Europe. Although official data state that approximately 1.5–2.0% of inhabitants in Slovakia, Czech Republic, and Serbia declare themselves as being of Roma nationality, the estimated real numbers are several times higher [6,7,8]. Roma communities tend to live in isolation from the rest of the population; they often concentrate in economically undeveloped regions and live in segregated localities and settlements [6,9]. Unsatisfactory housing and sanitary conditions and poor availability of services are characteristic features of such settlements. Compared to the majority, Roma often show a lower education level and higher rates of unemployment and poverty, factors considered as aggravating their hygienic and health conditions [10]. In general, Roma is considered to be a socio-economically disadvantaged population with worse health status and more frequent incidence of infectious diseases than the rest of the population.

Data on the occurrence of infectious diseases in the Roma minority are scarce, and only a few papers have concerned themselves with the incidence of parasitic diseases in this ethnicity. This study aimed to find out the seroprevalence of *Trichinella* and *Echinococcus* infections in the Roma population of segregated settlements and to compare it with the seropositivity of the non-Roma population of eastern Slovakia. Biochemical blood parameters, blood count, and clinical symptoms described in the questionnaire are also reported.

## 2. Materials and Methods

### 2.1. Serum Samples and Data Collection

The samples and data obtained during the HepaMeta project carried out in 2011 were used for the study. The project followed the principles of a community-based study, and the methodology was described previously by Madarasová Gecková et al. [11]. The monitored population consisted of Roma inhabitants of segregated settlements in the Košice region (eastern Slovakia). Non-Roma inhabitants of the same region (catchment area) were used as a control group.

Altogether, 823 respondents were involved and divided into two groups. The group of Roma respondents comprised 429 persons (148 men and 281 women), and 394 non-Roma subjects (182 men and 212 women) constituted the control group. Participants could not suffer from signs of acute illness, and their age varied between 18 and 55 years.

Aside from serum samples, data on the health status, education, socio-demographic and living conditions, and economic situation were collected from all respondents by questionnaire form.

The study was approved by the Ethics Committee at the P.J. Šafárik University in Košice. The study was performed under the anonymous conditions and all respondents signed informed consent before the participation.

### 2.2. Detection of Antibodies to Trichinella spp. and Echinococcus spp.

An indirect enzyme-linked immunosorbent assay (ELISA) was used for the first serological screening. *Trichinella spiralis* larval somatic antigen (TsAg) [12], *E. multilocularis* somatic antigen (EmAg) [13], and *E. granulosus* antigen B (AgB) [14] were used for the detection of antibodies to *Trichinella*, *E. multilocularis*, and *E. granulosus.* ELISA tests were performed as described previously by Reiterová et al. [15] with some modifications. Serum samples of patients with confirmed *Trichinella*, *E. multilocularis*, and *E. granulosus* infection and negative sera were used as controls. The cut-off was calculated for each antigen according to the optical density (OD) of 40 healthy controls (sera of people without clinical signs of any disease). The cut-off value was determined as the average of the negative control panel plus four standard deviations (SD).

ELISA-positive sera were subsequently tested using the Western blot (WB) method according to Reiterová et al. [15], with some modifications. Excretory/secretory antigen of *T. spiralis* [12], EmAg and sheep hydatid fluid antigen (HF) were separated on 12% SDS (sodium dodecyl sulfate) polyacrylamide gel under reducing conditions and transferred on a nitrocellulose membrane in tris-glycine buffer. Sample and control sera (diluted 1:50) were applied on the nitrocellulose strips and incubated for 1.5 h at 37 °C. Afterwards, the strips were incubated for an hour at 37 °C temperature with anti-human IgG peroxidase conjugate (Sigma-Aldrich, St. Louis, MO, USA) diluted at 1:500. The bands were visualized using 4-chloro-1-naphthol and 0.03% hydrogen peroxide. The banding patterns were compared with positive and negative controls and the protein molecular weight marker. WB was used as a confirmatory test, and only samples positive by both ELISA and WB were considered seropositive.

### 2.3. Statistical Analyses

Prevalence of antibodies analyzed to *Trichinella* spp. and *Echinococcus* spp. is described as relative frequency with 95% confidence interval (95% CI). Since there was minimal prevalence of seropositivity defined by ELISA cut-off or positive WB results we decided to analyze the quantitative values of circulating antibodies in relation to sociodemographic risk factors. As there was no reference standard available at the time of post hoc analysis, antibody levels are represented relatively as OD values of samples, which correspond linearly to the standard nomogram curve. Variables were analyzed using the *t*-test and ANOVA (Analysis of Variance) test, and univariate linear regression was used for analyses of infection risk factors. Two-sided *p* value < 0.05 was considered statistically significant.

### 2.4. Ethical Approval

Approval for the study was obtained from the Ethics Committee of the Faculty of Medicine at P.J. Šafárik University in Košice (No. 104/2011). Study was performed in accordance with the ethical standards as laid down in the Declaration of Helsinki of 1975 and revised in 2008. Participation in the study was voluntary and anonymous and informed consent was obtained prior to the medical examination.

## 3. Results

### 3.1. Characteristics of Analyzed Roma Population

As mentioned above, the data on the Roma and non-Roma populations were obtained and analyzed previously within the HepaMeta project. The results of baseline parameters of the involved populations showed significant differences in the life-style of the majority and the Roma minority in Slovakia and were published by Antolová et al. [16]. According to the mentioned analyses, Roma were more often unemployed, had achieved a lower level of education and more often suffered from poverty (characterized as the inability to cover common living expenses).

In the presented study, the average age of respondents in both involved groups was similar, 34.7 ± 9.1 in the Roma minority and 33.6 ± 7.4 in the majority population. Out of 429 Roma, 42.7% did not have access to drinking water; 52.9% did not have a sewage system; 49.0% did not have a toilet; 49.4% did not have a bathroom; and 17.2% had no electricity in their houses.

### 3.2. Seropositivity of Trichinella Infection

Out of 823 examined samples, only three sera showed seropositivity to *Trichinella* in the ELISA test. Subsequent WB confirmed seropositivity in two (0.24%; 95% CI 0.01–0.09) samples. Both positive sera came from Roma participants; thus, seropositivity calculated in Roma ethnicity reached 0.47% (95% CI 0.01–1.80) (Table 1). Both positive persons were women, age 44 and 35 years, respectively. They were unemployed, with an incomplete elementary education, and lived in brick houses with electricity and sanitary equipment (toilet, bathroom, and sewage system). Both women suffered from headache, cough, and fatigue, but only one reported muscle pain. Their blood count and biochemical blood markers (glucose, creatinine, albumin, AST (Aspartate aminotransferase), ALT (Alanine aminotransferase), GMT (Gamma-glutamyl transferase), cholesterol, triacyglycerols, etc.) were within physiological range.

Analysis of the relative circulating antibody levels (ELISA OD values) showed no significant difference between the Roma and the non-Roma participants (*p* = 0.968) (Table 2). Therefore, subjects were not divided into Roma and non-Roma categories in subsequent risk factor analyses. Univariate linear regression did not show any correlation between the analyzed factors (age, gender, education level, unemployment, number of people in a house, and lack of electricity and drinking water in the household). Linear regression showed some correlation between relative levels of circulating antibodies and the lack of a sewage system (*p* = 0.007) and toilet (*p* = 0.027) in the house (Table 3), but we do not suppose any relevant significance of this factor.

### 3.3. Seropositivity of Echinococcus multilocularis and Echinococcus granulosus Infection

ELISA seropositivity to *E. multilocularis* was recorded in six persons (0.73%), and five (0.61%) respondents were seropositive to *E. granulosus.* None of the seropositive samples were positive to both antigens. Consequent Western blot examination confirmed the presence of antibodies to *Echinococcus* spp. in one participant (0.12%; 95% CI 0.00–0.08). Unfortunately, the Western blot pattern of the sample did not allow distinguishing whether it was positive to *E. multilocularis* or *E. granulosus*. The positive participant belonged to the Roma community; therefore, seropositivity in the Roma minority was 0.23% (95% CI 0.00–1.45) (Table 4). According to the questionnaire, the positive person was a 40-year-old Roma woman who suffered from headache and back pain. She lived in a brick house without a bathroom, toilet, or sewage system, and used wood as a heating material. Clinical examination revealed hypertension (177/100 mmHg), but parameters of blood count and biochemical markers were within physiological range. Unfortunately, as the study was fully anonymous, it was impossible to contact the positive person to verify the results of the serology and to confirm or refute the diagnosis of echinococcosis.

As mentioned above, no significant difference in relative levels of circulating antibodies to *E. multilocularis* and *E. granulosus* between the Roma and non-Roma participants was present (*p* = 0.658 and 0.088, respectively) (Table 2 and Table 3). Therefore, subjects were not divided to Roma and non-Roma categories in subsequent risk factor analyses. Univariate linear regression did not show any correlation between the analyzed factors (age, gender, education level, unemployment, number of people in a house, and lack of electricity and sanitary equipment in the household).

## 4. Discussion

*Trichinella* spp., as well as *E. multilocularis* and *E. granulosus* s. l. are important zoonotic parasites threatening human health. Moreover, alveolar echinococcosis is considered one of the most dangerous human helminthoses as it can cause the death of untreated patients within 10 to 15 years after diagnosis [17]. All three species infect people via oral route and were assigned to the second (*E. granulosus*), third (*E. multilocularis*), and seventh (*Trichinella* spp.) positions on the list of top 10 most important food-borne parasites as released by the Food and Agriculture Organization of the United Nations (FAO) and World Health Organization (WHO) organizations in 2014 [18]. Pigs and wild boars are regarded as the most common source of human *Trichinella* infection and have also been confirmed as the source of the majority of human outbreaks in Slovakia [3]. The predominant species circulating in the territory of Slovakia is *Trichinella britovi* [19], which is also the most common causative agent of human outbreaks [3]. The course and severity of human infection depends on the *Trichinella* species involved, the infective dose and the phase of the infection. Most often, the infection is asymptomatic or connected only with mild symptoms. It is supposed that clinical signs appear after the ingestion of 100–300 infective larvae, and consumption of 1000–3000 larvae can cause severe trichinellosis [20,21]. Clinical symptoms of the disease include diarrhoea, nausea, vomiting, fever, facial edema, muscle pain, weakness, dyspnoea, cough, and headache [22]. In the presented study, both *Trichinella* seropositive participants suffered from headache, cough, and fatigue, while only one positive person reported muscle pain. Similarly, Ondriska et al. [23] described a mild course of trichinellosis during an outbreak of disease after the consumption of smoked sausages containing meat from an infected dog. Three out of 73 patients did not describe any sign of infection and 53 persons suffered from muscle pain. Later, during the outbreak in eastern Slovakia in 2008, muscle pain appeared in 9 out of 10 infected patients, and 6 and 2 patients, respectively, reported fatigue and headache [21]. According to the aforementioned data, we can suppose that both women presented in this study were infected with low dose of *Trichinella* larvae which caused the mild or even abortive course of the disease. Although univariate linear regression showed some correlation between relative levels of circulating antibodies and lack of sewage system and toilet in the house, we do not attach any real significance to this factor. *Trichinella* infection in men is connected with the consumption of undercooked meat and meat products, thus the lack of a sewage system in the house should not influence the possibility of being infected.

Echinococcosis, especially alveolar echinococcosis, is a life-threatening disease with a long incubation period and unspecific clinical symptoms. The infection is often recorded by chance, during the diagnostic process or surgery, due to other pathological findings. Serological tests are recommended not only as the method of choice in suspected cases, but also for the screening of infection in exposed groups (hunters, farmers, etc.) or in epidemiological studies [24]. Screening programs offer the possibility of detecting the infection in an earlier stage and subsequently better a prognosis of the disease. For accurate diagnosis of echinococcosis, a combination of serological tests is recommended [24,25]; therefore, in the presented study, the results of the ELISA method were verified by the more specific Western blot method. WB confirmed seropositivity to *Echinococcus* in one Roma woman. Although due to the anonymity of the study, it was not possible to perform the confirmatory imaging examination we can assume at least the contact of the positive person with the etiological agent of the disease. It is supposed that seropositivity to *Echinococcus* in humans indicates exposure to the parasite before disease development or without the establishment of the infection, as some of seropositive people show resistance to alveolar echinococcosis (AE) and do not develop visible hepatic lesions [17,26].

In Slovakia, more than 60 AE cases have been confirmed since 2000. While only a few cases (1–3) were reported every year between 2000 and 2011 [27], there has been an increase in the number of patients recorded since 2012, with up to 10 cases recorded every year. The majority, 43 out of 63 patients confirmed at the Institute of Parasitology SAS (Slovak Academy of Sciences) came from endemic, norther regions of Slovakia (Prešov, Žilina, and Trenčín regions), while the incidence of human AE in Eastern Slovakia was lower; only four patients came from the Košice region. One of the patients was of Roma nationality and came from a village in eastern Slovakia [28]. A study on the occurrence of *E. multilocularis* in red foxes performed between 2000 and 2010 showed the endemicity of northern regions of the country, where the prevalence of the parasite exceeded 40%, or even 50% and 60% in some districts. On the other hand, only 18.4% prevalence of the tapeworm was recorded in the Košice region located in the eastern part of the country [29]. Human cystic echinococcosis occurs in Slovakia sporadically; two or three confirmed cases are reported every year [4].

In general, studies on the Roma population show a higher prevalence of some parasite species in Roma than in the non-Roma population. In one study, Roma children from a segregated settlement with poor hygiene conditions, without a drinking water supply, or a sewage system were compared with non-Roma children from a neighboring village with higher hygienic standards and the municipal water supply and sewerage system available, and the difference in the prevalence of intestinal parasites was much more obvious, 50.4% in Roma vs. 0.0% in non-Roma children [30]. Similarly, Štrkolcová et al. [31] found eggs or oocysts of intestinal parasites in 46 (76.7%) of the children from the settlement, while only 2 (9.5%) children from the town were positive. In a previous study of Antolová et al. [16], Roma respondents were significantly more often seropositive to *Toxocara* (22.1%) than non-Roma participants (1.0%). Nevertheless, the results presented herein revealed that despite undoubtedly worse hygienic conditions in the segregated settlements, there were no significant differences in the prevalence of antibodies to *Trichinella* and *Echinococcus* spp. between inhabitants of Roma settlements and the majority population.

Human cases of *Trichinella* infection are primarily connected with the consumption of undercooked meat and meat products. In Roma cuisine, cooking and roasting meat are the most typical forms of meat preparation [32,33]. Therefore, even though the consumption of meat from dead animals occurs within the Roma minority, proper boiling or roasting prevents the spread of some food-borne diseases, including trichinellosis. The predominant source of *Echinococcus* spp. infective eggs in Slovakia are wild carnivores that contaminate their natural habitats. Although, Roma people very often live in poor hygienic conditions, together with high number of domestic animals, mainly dogs, in a small area, the study did not reveal a higher infection risk of echinococcosis than in the majority population. The study was performed in eastern Slovakia, where the prevalence of *E. multilocularis* is lower than in other parts of the country; therefore, we can assume the lower infectious pressure of the environment. The risk of being infected is probably more connected to the contact with a contaminated environment than with life in segregated settlements.

## 5. Conclusions

The study revealed that the undoubtedly worse living and hygienic conditions in segregated Roma settlements did not influence the seropositivity of *Trichinella* and *Echinococcus* infections in this minority. Nevertheless, the lack of hygiene, limited access to health care, and the high number of domestic animals can increase the risk of the spread of other infectious diseases. Therefore, the authorities should pay attention to measures aimed at screening and prevention of communicable diseases in Roma people.

## Figures and Tables

**Table 1:** ELISA (Enzyme-linked immunosorbet assay) and Western blot (WB) seropositivity to *Trichinella* spp. in Roma and non-Roma population of Eastern Slovakia.

Parameter	ELISA Positivity *N* (%)	WB Positivity *N* (%)
Roma population (*n* = 429)	3 (0.70)	2 (0.47)
Non-Roma population (*n* = 394)	0 (0.00)	0 (0.00)
Total (*n* = 823)	3 (0.36)	2 (0.24)

*N*—number of positive; *n*—number of participants per group.

**Table 2:** Results of statistical analyses (*t*-test, Analysis of Variance (ANOVA) and univariate linear regression) of results of ELISA method (optical density (OD) values) in Roma and non-Roma participants of study.

OD Values	Roma vs. Non-Roma Participants					
	Mean	Standard Error of Mean	Mean	Standard Error of Mean	*p*-Value	Linear Regression
*Trichinella* spp.	0.127	0.004	0.127	0.004	0.968	0.001
*E. multilocularis*	0.140	0.006	0.137	0.004	0.658	−0.015
*E. granulosus*	0.157	0.004	0.168	0.005	0.088	0.060

**Table 3:** Relationship between relative levels of antibodies (OD values) and sociodemographic risk factors in Roma and non-Roma participants of study.

Parameter	*Trichinella*		*E. multilocularis*		*E. granulosus*	
	SRC	*p*-Value	SRC	*p*-Value	SRC	*p*-Value
Gender	0.011	0.747	0.043	0.220	−0.006	0.865
Age	−0.044	0.216	0.027	0.450	0.048	0.175
Unemployment	0.030	0.392	0.028	0.424	−0.054	0.120
Education	−0.007	0.839	−0.031	0.377	0.064	0.068
Missing sewage system	0.079	0.007	−0.170	0.630	0.042	0.226
Missing drinking water piping	0.036	0.298	−0.006	0.87	0.033	0.348
Missing toilet	0.059	0.027	−0.027	0.440	0.006	0.853
Missing electricity	0.005	0.887	0.027	0.447	0.036	0.302
Payment problems	0.061	0.081	−0.006	0.875	−0.005	0.879

SRC—Standard regression coefficient.

**Table 4:** ELISA and WB seropositivity to *Echinococcus* spp. in Roma and non-Roma population of Eastern Slovakia.

Parameter		ELISA Positivity *N* (%)	WB Positivity *N* (%)
Roma population (*n* = 429)	*E. multilocularis*	4 (0.93)	1 (0.23)
	*E. granulosus*	2 (0.47)	
Non-Roma population (*n* = 394)	*E. multilocularis*	2 (0.51)	0 (0.00)
	*E. granulosus*	3 (0.76)	
Total (*n* = 823)	*E. multilocularis*	6 (0.73)	1 (0.12)
	*E. granulosus*	5 (0.61)	

*N*—number of positive; *n*—number of participants per group.
